# Evaluation of MyTeen – a SMS-based mobile intervention for parents of adolescents: a randomised controlled trial protocol

**DOI:** 10.1186/s12889-018-6132-z

**Published:** 2018-10-26

**Authors:** Joanna Ting Wai Chu, Robyn Whittaker, Yannan Jiang, Angela Wadham, Karolina Stasiak, Matthew Shepherd, Chris Bullen

**Affiliations:** 1 The National Institute for Health Innovation, School of Population Health, University of Auckland, Auckland, New Zealand; 2 Department of Psychological Medicine, Faculty of Medical and Health Sciences, University of Auckland, Auckland, New Zealand; 3 School of Counselling, Human Services & Social Work School of Counselling, University of Auckland, Auckland, New Zealand

**Keywords:** Parenting intervention, MHealth, Adolescent, Mental health, Prevention

## Abstract

**Background:**

Parents play an important role in the lives of adolescents and efforts aimed at strengthening parenting skills and increasing knowledge on adolescent development hold much promise to prevent and mitigate adolescent mental health problems. Innovative interventions that make use of technology-based platforms might be an effective and efficient way to deliver such support to parents. This protocol presents the design of a randomised controlled trial to investigate the effectiveness of a SMS-based mobile intervention (MyTeen) for parents of adolescents on promoting parental competence and mental health literacy.

**Methods:**

A parallel two-arm randomised controlled trial will be conducted in New Zealand, aiming to recruit 214 parents or primary caregivers of adolescents aged 10–15 years via community outreach and social media. Eligible participants will be allocated 1:1 into the control or the intervention group, stratified by ethnicity. The intervention group will receive a tailored programme of text messages aimed at improving their parental competence and mental health literacy, over 4 weeks. The control group (care-as-usual) will receive no intervention from the research team, but can access alternative services if they wish, and will be offered the intervention programme upon completion of a 3-month post-randomisation follow-up assessment. Data will be obtained at baseline, post intervention (1-month), and 3-month follow up. The primary outcome is parental competence assessed by the Parental Sense of Competence Scale at 1-month follow up. Secondary outcomes include: mental health literacy; knowledge of help-seeking; parental distress; parent-adolescent communication; and programme satisfaction.

**Discussion:**

To our knowledge this is the first randomised controlled trial on the effectiveness of delivering a parenting support intervention for parents of adolescents solely via a SMS-based mobile intervention. If effective, it could have great potential to reach and support parents of adolescents.

**Trial registration:**

Australian New Zealand Clinical Trial Registry (ACTRN12618000117213) Registered on 29/01/2018.

## Background

Depression is a leading cause of morbidity in adolescents, and a major risk factor for suicide, the second most common cause of death in this age group [1]. Depression has a high rate of relapse and commonly starts in adolescence. The effect of depressive disorder is pervasive and affects not only function but overall development. Depressive disorder is associated with various adverse outcomes, including lower educational attainment, social dysfunction, substance use, attempted and completed suicide [2, 3]. Co-morbidity is high, with nearly 60% of those with major depressive disorder having a life time occurrence of another psychiatric disorder [3]. In New Zealand, depressive disorder is the most common mental health disorder amongst adolescents, with prevalence rates of 4–8% at the age of 15 increasing rapidly to 17–18% by 18 years of age [4]. Recent New Zealand representative study showed that rates of depression for Māori (the indigenous people of New Zealand) high school students (at 13.9%) were comparable to New Zealand European young people (12.1%). Of concern is the high rate of reported clinical depressive symptoms (18.3% for Māori girls and 8.7% for Māori boys) measured by the Reynolds Adolescent Depression Scale – Short Form (RADS-SF) [3, 4]. Evidence from the past two decades suggests that prevention programmes reduce the incidence of mental health problems [5]. The importance of preventive interventions has been emphasized by numerous expert panels [6, 7]. The serious developmental consequences of adolescent depression, the associated treatment challenges and the high costs once it has developed, underscore the need for interventions aimed at prevention [8, 9]. Current clinical practice, generally limited to treating depression in its acute phase, fails to alleviate the disease burden in a significant way at the population level [10].

### Parents as an important target for prevention and early intervention

A critical factor in an adolescent’s outcome is the extent to which their parents are responsive and supportive to their developmental needs and skilled in managing their child’s behaviour [11, 12]. Research from the field of developmental psychopathology links a number of family risks and protective factors (e.g. quality of parent-child relationship, parental self-efficacy, parental adjustment, and parenting practices) to adverse mental health outcomes in adolescents [11, 13–15]. Stressful family environment (e.g., frequent arguments, escalating hostility, criticism, or anger) can undermine adolescents’ coping resources and increase their risk for depression [2, 14, 15]. Parents play a significant role in mediating risk for youth exposed to high levels of adversity [15, 16] and the protective role of positive parenting holds, irrespective of socio-economic status and levels of neighbourhood distress [17].

Grounded in evidence-based approaches, including social learning models, self-regulation theory, and cognitive behavioural therapy, parenting programmes aimed at strengthening parenting skills and increasing knowledge on adolescent development have led to significant improvement in parent-adolescent relationships and a reduction in adolescent mental health problems [6, 18–22]. Studies have reported that parent’s acknowledgement of their child’s depression is associated with adolescent’s readiness (i.e., perceptions about viewing depression as a problem, understanding the symptoms, and wanting to get help) to seek professional help [23]. Young people themselves also see parents as one of the most important sources of support for receiving help on mental health problems [24]. The prevention and early intervention efforts that effectively up skill parents thus have great potential in preventing depression in youths.

### Barriers to accessing services

Even when promising programmes are available to support parents, engaging families can be challenging, with engagement rates as low as 10% for supplemental parenting training when added to individual treatment for depressed adolescents [25]. Traditional face-to-face intervention is resource intensive and, depending on the setting, can be difficult to implement on a large scale with limited reach to some population groups. Primary logistic barriers for accessing services include lack of time, cost, transport, rural isolation, scheduling conflicts and competing demands. These are further compounded by other barriers including perceived stigma, shame, scepticism, distrust of the system/professionals, and low mental health literacy, including poor awareness of signs/symptoms and resources [26–29]. While home visits are known to be effective for reaching parents, there are limitations to resourcing and some families are also resistant to these [30, 31].

In short, sole reliance on traditional modes of intervention is insufficient for the level of need and demand. Interventions that make use of technology-based platforms and that reach parents, might be at least as effective and potentially more efficient and acceptable in addressing adolescent mental health problems.

### Mobile health (mHealth) intervention in supporting parents of adolescents

MHealth interventions have great potential for public health impact because of their broad reach and convenience [26]. mHealth offers a wide range of potential benefits over traditional approaches, such as (1) programmes can be delivered anywhere at any time, and for extended periods, facilitating regular communication and behavioural maintenance; (2) support via messages or notifications are sent directly to people in a time-sensitive manner, which means the program can be designed to fit in with the individual’s lifestyle and provide prompts at the most appropriate times (3) program are more proactive (initiated by the service) than traditional services, which often require action or attendance by the participant before they can impart information or provide support; (4) they are flexible, and can be personalised and tailored to specific cultural, age group, and health needs; (5) reach is increased because the barriers of face-to-face contact (such as time, cost and travel) are removed and; (6) disparities in access across the socio-economic status gradient are decreased due to the high penetration of mobile phones across all groups [26]. Opinion surveys indicate that parents who are interested in family programmes have a stronger preference for mhealth interventions over face-to-face delivery [32, 33].

While text messaging may not be considered a ‘novel’ mobile phone application, globally, it remains the most widely used [26]. It is also inexpensive to develop and deliver and it requires minimal technological ‘know how’. Text messaging also requires very basic, low-cost phones (to receive and send messages), which reduces potential socioeconomic disparity of access (‘digital divide’). Text messaging programmes have successfully promoted parenting behaviour change in a number of important domains for parents of young children: decreasing the likelihood of abuse and neglect, increasing childhood vaccinations, and encouraging healthy pregnancies [34–36]. Studies have reported that text messaging interventions were well received by parents of various populations including those that are socially deprived [36]. While there is evidence to suggest the feasibility and effectiveness of SMS mobile-based interventions to address other health issues [34, 35, 37], its application and impact among adolescent parent populations are unknown.

#### Rationale for research

The prevalence of mental health problems in youth is substantial; and efforts aimed at strengthening parenting skills and increasing knowledge on adolescent development hold much promise to prevent and mitigate adolescent mental health problems. To date there have been no reported investigations on the efficacy of delivering a parenting support intervention for parents of adolescents via a mobile-based intervention. We evaluate the effectiveness of a SMS-based mobile intervention for parents of adolescents on promoting parental competence and mental health literacy.

#### Objective

The aims of this trial is to determine whether a 4-week SMS-based mobile intervention (MyTeen) for parents of adolescents can lead to:

1. Early improved parental sense of competence (primary outcome)
2. Continued improvement in parental sense of competence
3. Improved parental knowledge on depression
4. Improved knowledge on mental health seeking
5. Reduced parental stress
6. Improved quality in parent-adolescent communication

## Methods

### Design

This study is a parallel two-arm randomised controlled trial (RCT). Eligible parents/primary caregivers (hereafter referred to as parents) will be randomly allocated to the MyTeen intervention programme or care-as-usual condition. Data will be obtained from all participants at baseline, 1 month (end of intervention phase), and 3 months post randomisation. The study has been approved by the University of Auckland Human Participants Ethics Committee (UAHPEC) and is registered with the Australian New Zealand Clinical Trial Registry (ACTRN12618000117213).

### Participants

A total of 214 parents residing in New Zealand will be recruited. Parents will be eligible for inclusion in the study if they indicate at screening that they have an adolescent child aged between 10 and 15 years of age; have access to a mobile phone; are not currently receiving any professional assistance for their own and/or child’s mental health problems; possess adequate knowledge of the English language; and are willing to participate in the study and provide follow-up information at scheduled points of the study. Only one parent from each household will be invited to participate. Parents that do not meet the inclusion criteria will be excluded from the study. In addition, parents that show high level of stress as reported by the Parental Stress Index (i.e., ≥ 72) at screening will be excluded and directed to professional services.

### Setting

This study will be conducted nationwide in New Zealand. An outreach approach will be conducted to promote the study. In additional to social media channels, potential participants may also hear about the programme through word of mouth, and flyers distributed through schools, community organisations such as sports, cultural clubs and faith communities.

### Study procedures

Potential participants can either call or text a phone number to speak with a research assistant or leave their contact details via email. The research assistant will contact the participant and explain the study, obtain verbal consent to ask screening questions to ascertain their eligibility for the study. Eligible participants who indicate interest will be sent an email with the participant information sheet and consent form. A separate email will be sent from the study database containing a link to the first online questionnaire. Before they complete the questionnaire they will be asked to provide e-consent. Participants will then be directed to complete the baseline assessment. Randomisation will be performed upon completion of the baseline assessment, and participants will be notified via email which group they have been allocated to. Those that are randomised into the intervention group will receive the MyTeen programme the day following the notification email. Participants that are randomised into the care-as-usual group will receive no intervention from the research group. At 1-month post randomisation, participants in both groups will receive an email directing them to complete the 1-month follow up assessment. Another email will be sent at 3 months for participants to complete the 3-month follow up assessment. Reminder emails and/or texts will be sent if participants do not complete the assessments. A follow-up phone call will be made to the participant if the online assessment is not completed after two reminders. Upon completion of the 3-month follow up, the MyTeen programme will be offered to participants in the control group. Each participant will receive a $NZD20 petrol voucher in appreciation of his or her time given to the study.

### Randomisation

Parents (*N* = 214) who have completed baseline assessment and fulfil all entry criteria will be randomised at a 1:1 ratio to either the MyTeen intervention group or the control (care-as-usual) group. The randomisation sequence will be generated by the trial statistician using block randomisation with variable block sizes of 2 or 4, and stratified by Māori, Pacific, and non-Māori/non-Pacific ethnic groups. The final randomisation lists will be computer-generated and concealed in secure study database until the point of randomisation.

### Blinding

Due to the nature of the intervention, it will not be possible to blind participants or research staff to the allocated treatments.

### Study intervention

Intervention: All intervention participants will receive MyTeen, a tailored programme of text messages (SMS) via their mobile phone. The messages provide instructional, informational, and emotional support. These include evidence-based information (adapted from the Parenting Strategies Program) on the nature and symptoms of depression, understanding treatment options, strategies to improve parent-child communication, parent self-care, and useful links to resources. Formative work (i.e. focus groups) was conducted with parents to ensure that the content, intensity, and duration of the intervention were appropriate and feasible. One daily text message will be sent to participants over 4 weeks. Participants will receive all the text-messages free of charge.

Care as usual: Participants allocated to the care-as-usual control group will receive no intervention from the research team, and can access alternative services if they so desire. On completion of the 3-months follow up assessment, participants will be offered the MyTeen programme.

### Measures

Table 1 shows the schedule of outcome assessments measured at various time points. At baseline assessment, demographic data including age, sex, marital status, ethnicity, education level, employment status, household income, family structure, child’s age, child’s sex, and child’s ethnicity will be collected.

**Table 1:** Schedule of outcome assessments measured at various time points

Timing		0 Week	1 month	3 months
Description	Screening	Baseline data collection + Randomisation	Follow-up data collection	Follow-up data collection
General data				
Verbal informed consent	✓			
E-consent		✓		
Eligibility	✓			
Randomisation		✓		
Age, sex, ethnicity		✓		
Socioeconomic position		✓		
Family structure		✓		
Child information		✓		
Contact details	✓		✓	✓
Form Assessments				
Parental Competence (PSOC)		✓	✓	✓
Knowledge of mental health issue (Parent knowledge of depression)		✓	✓	✓
Knowledge of mental health help seeking (MHLS)		✓	✓	✓
Parental Distress (PSS)	✓		✓	✓
Parent-adolescent communication (PASC)		✓	✓	✓
Program satisfaction (Intervention Only)			✓	
Exit Interview				
Feedback			✓	

#### Primary outcome

The primary outcomes measure will be assessed at 1 month post randomisation by the Parental Sense of Competence (PSOC) [38] which measures parental self-esteem on two dimensions: Satisfaction and Efficacy. Satisfaction examines the degree to which parents’ feels frustrated, anxious, and motivation in the parental role, while the Efficacy reflects parents’ competence capability levels and problem-solving abilities in their parental role. The constructs of satisfaction and efficacy are closely linked with a host of positive family interactions as well as with positive child development [38]. The total score of PSOC is calculated as the sum of 17 items, and has a possible range of 17 to 102. The PSOC appears to be sensitive to changes resulting from brief parenting support [39]. In a New Zealand sample, the scale has had good internal reliabilities of 0.81 and 0.88 for the satisfaction and efficacy subscales, respectively [40].

#### Secondary outcomes

Secondary outcomes include the PSOC scale at 3-month follow up.

Knowledge of mental health issues in youth will be measured by a 7-item scale developed by Fox [41] at 1- and 3-month follow up. Participating parents or caregivers will be asked to rate the extent to which they agree or disagree with the items. The responses from each item are recoded such that each correct response gives a score of 1 and each incorrect response is given a score of 0. The scores are then summed to create the parent knowledge score which ranges from 0 to 7, where a higher score indicates greater knowledge of depression.

Knowledge of where to seek mental health information will be measured by the subscale on knowledge of where to seek information from the Mental Health Literacy Scale (MHLS) [42] at 1- and 3-month follow up. The subscale consists of 4-items, rated on a 5-point scale, ranging from *strongly disagree* to *strongly agree.* The scale has demonstrated good internal and test-retest reliability, and scores are significantly correlated with help seeking intentions.

Level of parental distress will be measured at 1- and 3-month follow up by the Parental Stress Scale, (PSS) [43] which consist of 18 items rated on a 5-point scale from ‘strongly disagree’ to ‘strongly agree’ to generate a total score. The scale has good internal reliability (.83), and test-retest reliability (.81).

The quality of parent-adolescent communication will be measured at 1- and 3-month follow up by the Parent-adolescent Communication Scale, (PACS) [44] consisting of 20 items that generates a total score and two subscale score (open family communication and problems in family communication). The scale has good internal reliabilities for both sub scales (0.87 and 0.78, respectively) and test-retest reliabilities (0.78 and 0.77, respectively). Programme satisfaction will be measured at 1-month follow up and completed by the intervention group only.

Qualitative data: Exit interviews will be done with a subset of participants to capture participants’ experience with the programme.

### Sample size calculation

We aim to recruit 214 participants in total (*n* = 107 per randomised group; one parent per household; 30% Māori, 30% Pacific). This sample size will provide 80% power at *p* = 0.05 to detect a group difference of 2.5 in the Parenting Sense of Competence scale (PSOC) score at 1-month follow up (SD = 5.8), and allowing for an estimated 20% loss to follow up.

### Data analyses

Data from the RCT will be entered into a RedCap database, and following cleaning and datalock, extracted into SAS version 9.4 (SAS Institute Inc., Cary, NC, USA) for analysis. All data analyses will be specified a priori in a statistical analysis plan. No interim analysis will be undertaken. *Baseline characteristics:* Baseline data collected from all participants will be summarised by treatment group, overall and by ethnicity (Māori, Pasifika and non- Māori non-Pacific). Continuous variables will be presented as numbers observed, means and standard deviations. Categorical variables will be presented as frequencies and percentages. Since any differences between randomised groups at baseline could only have occurred by chance, no formal significance testing will be conducted. *Intervention effects:* Analysis will be carried out on an intention-to-treat basis including all randomised participants. Sensitivity analyses will be undertaken to determine the impact of missing data (if any) under different assumptions. Primary and secondary outcomes will be first summarised descriptively by treatment group at each time point. Generalised linear mixed models will be used to assess the overall intervention effect on each outcome at 1 and 3 months, adjusting for baseline outcome value and ethnicity (stratification factor). Repeated measures on the same participant will be taken into account in analysis using a random subject effect. Model-adjusted estimates of group difference and 95% confidence intervals will be reported with associated *p*-values. All statistical tests will be two-sided at 5% significance level. Subgroup analysis by ethnicity will be conducted to evaluate the consistency of intervention effects across ethnic groups, if the recruitment targets are met.

### Reporting of results

The CONSORT 2010 statement will be followed as the guidelines for reporting parallel group randomised trials. The overall trial results will be communicated through presentations at national and international conferences, and articles in peer-reviewed scientific journals. Study participants will be informed about the trial results by being sent a plain language summary of the results. The general public will be informed about the trial via posting of the research findings on the University’s and other relevant websites. Academic papers and summary reports will be provided to funders.

## Discussion

This paper presents the design of a randomised controlled trial to investigate the effectiveness of a SMS-based mobile intervention for parents of adolescents. To the authors’ knowledge, there have been no reported investigations on the effectiveness of delivering a parenting support intervention for parents of adolescents via a SMS-based mobile intervention.

Studying the effects of this intervention is important as it aims to generate knowledge on the potential of a mHealth model of parenting support. If effective, the intervention can be easily scaled-up for national roll-out, and be adapted to enhance support for a diverse parenting population (e.g., parents of young children, parents of older adolescents, and various ethnic groups) on mental health issues. Our research will contribute to the goal of improving outcomes for families and youths by 1) increasing parental competence and mental health literacy to promote early recognition and appropriate help seeking behaviours; 2) providing a low-cost, sustainable parenting intervention with broad population reach; and 3) using technology to reduce disparities in utilisation of existing services, as mobile phones are used by all regardless of socioeconomic status or ethnicity.

### Limitations

This study has potential limitations. First, as a mhealth intervention, technology related issues may present barriers to intervention content delivery. To address this limitation, we have a strong technical development and support team to monitor and resolve any technology issues throughout the intervention period. Second, due to limited resources, only 1- and 3-months follow up are conducted with participants, therefore long term effects of the intervention are unknown. Nonetheless, the findings from the short-term outcome will provide preliminary data and inform future research. Finally, although multiple method and informant data sources are ideal for a test of effectiveness, all of the outcomes will be obtained by parents’ self-report. It is worth noting that findings from RCTs of parenting programmes have reported significant results across parent self-report, teacher report, and parent-child observation [45] suggesting that parent self-reports are consistent with other methods of measuring parent and child outcomes.

Despite the limitations, demand for mental health services far exceeds the current services offered [7, 46], and new approaches are therefore needed to enhance access to support parents of adolescents. MHealth interventions targeting parents of adolescents remains largely untapped, but these programmes may comprise a promising approach to preventing and mitigating adolescent mental health problems by widening reach, increasing population level impact, and reduce inequalities due to lack of access to existing services. This study will provide new knowledge about the effectiveness of a SMS-based mobile intervention for parents of adolescents. Information on uptake and adherence to this type of intervention will also be generated to inform future studies in support parents of adolescents.

## Abbreviations

MHealth: Mobile health
MHLS: Mental Health Literacy Scale
PACS: Parent-adolescent communication scale
PSOC: Parental Sense of Competence
PSS: Parental Stress Scale
RADS-SF: Reynolds Adolescent Depression Scale – Short Form
RCT: Randomised controlled trial
UAHPEC: University of Auckland Human Participants Ethics Committee
